# The Proper Scope of Legislation for Midwives

**Published:** 1900-02-24

**Authors:** 


### THE PROPER SCOPE OF LEGISLATION FOR MIDWIVES.

A Bill "to secure the better training of midwives and to regulate their practice" having been introduced into the House of Commons, and the second reading having been set down for next Wednesday, small as is the likelihood of the Bill being reached, it may be as well to urge that, whatever be done, the main object of undertaking legislation, the main excuse for introducing a Bill at all, namely, the protection of the poor from the evils associated with the employment of ignorant midwives, should not be overlooked. The days of class legislation are past and gone, and just as no opposition to the Bill on the ground of its interfering with the interests of the medical profession is likely to be listened to for a moment, so it is to be hoped that no attempt to turn the Bill into one for improving the status of the midwives will receive the slightest support. The sole excuse for undertaking legislation on the subject is not the desires of the doctors nor the ambitions of the midwives of the future, but the fact that the unregulated and uncontrolled position occupied by the midwives of the present involves certain well recognised dangers upon the mothers of England. The justice of the proposals made must then be measured entirely by their capacity for levelling up the bottom stratum of midwifery work, and for enforcing among the lowest class of midwives a higher standard of work than they have hitherto been accustomed to. And here is the weakness of the Bill, for while its provisions are such that it could be made an engine of the greatest utility, and that it could be so used as to do the greatest good, there is no security that it might not be used in quite a different way.

In discussing this problem let us try to get rid of the notion, which seems to have fixed itself so strongly in the minds of some of our profession, that midwifery is work which can only properly be done by those who have undergone a complete medical education. In essence the act of parturition is a normal function, and when performed by a healthy woman in a healthy way has no relation to a profession engaged in the treatment of disease, except that arising from the convenience of having help at hand during a process in which help may at any time be wanted. One of the last speeches made by the late Sir Richard Thorne Thorne at the General Medical Council was on this very subject. He said that "he felt very strongly that nothing could be more unworthy of the medical profession than to say—and it amounted to that—that no woman should go through the perfectly healthy, perfectly normal, and physiological process of natural labour without paying a doctor's fee, because to say that no person without full medical training should be allowed to attend these cases amounted to that." What, then, is the proper relation of the medical profession to midwifery? Surely it is the same as its relation to every other event in ordinary life, namely, that when accident or disease arises then the doctor is to be called in. Perhaps not all the interests, but certainly all the rights of the medical profession hinge upon preventing legal sanction being given to the treatment of cases of accident or disease, whether arising in the course of a confinement or not, by any but qualified medical practitioners. If the point is firmly grasped that disease is a matter for the doctors, and that the sort of women contemplated by the Bill should be those whose work it will be to attend ordinary cases only, and whose first duty it will be to send for help as soon as anything out of the ordinary way occurs, it becomes clear that no very long or expensive training should be demanded. We are afraid, however, that this is hardly the ambition of those who are pushing on the Bill, and there can be no doubt that one of the dangers involved in it is that the Midwives' Board, which is to be appointed under the Act, may take it into its head to demand a high qualification, and may thus wreck the very object for which legislation is demanded, namely, the provision *for the poor* of a better class of midwives than those on whom they have to depend at present. We have to think of the villages and the outlying country districts, where certain working women among their other duties attend their neighbours in childbirth. These are just the women whose doings should be controlled. But if the qualifications demanded by the Midwives' Board should prove prohibitive they will not become licensed, and one thing or the other will happen, either great hardship will be caused, or the law will be rendered inoperative in consequence of the magistrates, in the exercise of their common sense, refusing to convict. The utility of legislation from the point of view of the public and the justice of legislation from the point of view of the medical profession, hinge on the same thing, namely, that the measure shall be framed in the interests of the poor, and with the object of securing a certain standard of training and of conduct among all midwives, even those of the lowest class, rather then of giving status and position to quite a different class of midwives with whom the poor may have very little to do at all. Everything, however, is made to depend upon the Midwives' Board, and that is the weak spot of the Bill.

